# Indicators of hypercoagulability and recurrent venous thromboembolism in the elderly: rethinking age and thrombophilia

**DOI:** 10.1016/j.rpth.2023.100056

**Published:** 2023-02-15

**Authors:** Saskia Middeldorp

**Affiliations:** Department of Internal Medicine, Radboud university medical center, Nijmegen, The Netherlands

In the current issue of RPTH, Méan and colleagues [1] describe a cohort of elderly people who survived 1 year after the first episode of venous thromboembolism (VTE). They observed several laboratory indicators of hypercoagulability that were associated with recurrent VTE and mortality. The authors investigated a mix of established thrombophilias (ie, deficiencies of antithrombin, protein C and protein S, and antiphospholipid antibodies) as well as plasma levels of some coagulation factors (factor [F]VIII, FIX, and fibrinogen) and homocysteine. The authors conclude that 78% of the elderly patients in their cohort had ≥1 “laboratory thrombophilic risk factors” and that such factors potentially allow identification of a population at risk of worse clinical outcome.

There are several interesting aspects about the study that evolve around applicability of clinical practice and current thinking about thrombophilia and thrombophilia testing. At present, thrombophilia testing is most often considered in patients with VTE, particularly if they are young, have recurrent episodes, have thrombosis at unusual sites, or have a positive family history of the disease. Although such a strategy will lead to an increased yield of testing and contributes to etiologic insight, it has been debated for decades whether thrombophilia testing should alter management, and hence, whether testing should be performed at all. [2] In the upcoming evidence-based American Society of Hematology VTE Guidelines on thrombophilia testing, a modeling approach in the absence of direct evidence has led to (weak) recommendations that suggest performing testing in selected clinical scenarios where therapeutic consequences from a positive test result may arise for patients with VTE or individuals with a positive family history of VTE or inherited thrombophilia. [3] The present study suggests that the prevalence of established thrombophilias in older people is not materially different from that in younger populations with VTE. [2] This challenges the common approach to decide about thrombophilia testing on the basis of age. On the other hand, the risk of recurrent VTE also increases with age, and hence, the contribution of thrombophilia to reaching a threshold of recurrent VTE risk that tips the balance toward long-term anticoagulation may intuitively be less than in younger patients. Indeed, in a previous analysis of the same study cohort, the author showed that FV Leiden and prothrombin 20210A mutation did not increase the risk of recurrent VTE (FV Leiden subhazard ratio, 0.98; 95% CI, 0.35-2.77 and prothrombin G20210A mutation subhazard ratio, 1.15; 95% CI, 0.25-5.19). [4] However, the CIs around the point estimate do not exclude the consistently observed odds ratio of 1.4 for these mutations in a large systematic review. [5] In my view, the current study underlines that there is no reason to assess the risk of VTE recurrence in elderly patients differently than in younger patients.

It is important to note that a current standard practice test panel of inherited and acquired thrombophilias consists of FV Leiden; prothrombin 20210A mutation; and levels of antithrombin, protein C, and protein S to detect an inherited deficiency and antiphospholipid antibodies (Table). In this elderly population, the prevalence of antithrombin deficiency was higher than expected, and as the authors discuss, it is likely that these patients have an acquired antithrombin deficiency rather than an inherited form. Whether underlying conditions confound the association with recurrent VTE or mortality in the study cannot be excluded. Assessment of levels of coagulation factors and homocysteine is not standard in clinical practice, and calling these “thrombophilic risk factors” in the text and the tables (rather than “indicators of hypercoagulability”) may be confusing to the reader. Regardless of nomenclature and whether these factors belong in a thrombophilia panel or not, levels of coagulation factors are known to be associated with VTE risk and have a high degree of heritability. [6,7] Levels of FVIII as an inherited thrombophilia have been studied extensively. We previously showed that 40% of first-degree relatives of patients with elevated FVIII plasma levels also have levels of FVIII above the 75th percentile of the normal population. [8] Although hyperhomocysteinaemia has also been widely studied as a thrombophilia risk factor, it should be deleted from standard practice thrombophilia panels. The fact that there is no association between polymorphisms—that lead to hyperhomocysteinaemia with reduced folate or vitamin B6 or B12 status—and VTE [9] as well as the fact that correction of mild hyperhomocysteinemia does not reduce the risk of recurrent VTE [10] are strong arguments to assuming underlying conditions as the true risk factors for (recurrent) VTE. Importantly, the levels of coagulation factors VIII, IX, and XI all increase with age and therefore are likely candidates for consideration as age-specific VTE risk factors. Studying the effect of these laboratory indicators of hypercoagulability in older people is likely to provide mechanistic insights that will be relevant for the young too. [11]

**Table:** Thrombophilia tests

Standard practice thrombophilia panel	Not in standard practice thrombophilia panel	For etiologic research purposes
Inherited	MTHFR mutation	FVIII
FV Leiden	Homocysteine	FIX
Prothrombin 20210A mutation	FVIII	FXI
Antithrombin activity^a^		Fibrinogen
Protein C activity^a^		
Protein S activity^a^		
Acquired		
Lupus anticoagulant		
Anticardiolipin IgG		
Anticardiolipin IgM		
Anti–Beta2-glycoprotein I IgG		
Anti–Beta2-glycoprotein I IgM		

F, factor; Ig, immunoglobulin; MTHFR, methylenetetrahydrofolate reductase.

a Repeat if abnormal, consider acquired deficiencies, confirm inherited nature by family testing

In conclusion, in elderly patients with VTE, established thrombophilias are present and contribute to VTE risk. Therefore, rational thrombophilia testing in specific clinical scenarios should not exclude patients merely on the basis of being older. Elevated levels of various coagulation factors that are known to increase with age are associated with recurrent VTE and mortality. It is likely that the latter phenomenon, at least in part, explains the increased risk of first and recurrent VTE risk in older as compared with younger patients. However, testing for these factors is not relevant to clinical practice.
